# Distribution of Mammalian-Like Melanopsin in Cyclostome Retinas Exhibiting a Different Extent of Visual Functions

**DOI:** 10.1371/journal.pone.0108209

**Published:** 2014-09-24

**Authors:** Lanfang Sun, Emi Kawano-Yamashita, Takashi Nagata, Hisao Tsukamoto, Yuji Furutani, Mitsumasa Koyanagi, Akihisa Terakita

**Affiliations:** 1 Department of Biology and Geosciences, Graduate School of Science, Osaka City University, Osaka, Japan; 2 Department of life and Coordination-Complex Molecular Science, Institute for Molecular Science, Okazaki, Japan; 3 Department of Structural Molecular Science, The Graduate University for Advanced Studies (SOKENDAI), Okazaki, Japan; 4 PRESTO, Japan Science and Technology Agency, Saitama, Japan; NIH/NEI, United States of America

## Abstract

Mammals contain 1 melanopsin (*Opn4*) gene that is expressed in a subset of retinal ganglion cells to serve as a photopigment involved in non-image-forming vision such as photoentrainment of circadian rhythms. In contrast, most nonmammalian vertebrates possess multiple melanopsins that are distributed in various types of retinal cells; however, their functions remain unclear. We previously found that the lamprey has only 1 type of mammalian-like melanopsin gene, which is similar to that observed in mammals. Here we investigated the molecular properties and localization of melanopsin in the lamprey and other cyclostome hagfish retinas, which contribute to visual functions including image-forming vision and mainly to non-image-forming vision, respectively. We isolated 1 type of mammalian-like melanopsin cDNA from the eyes of each species. We showed that the recombinant lamprey melanopsin was a blue light-sensitive pigment and that both the lamprey and hagfish melanopsins caused light-dependent increases in calcium ion concentration in cultured cells in a manner that was similar to that observed for mammalian melanopsins. We observed that melanopsin was distributed in several types of retinal cells, including horizontal cells and ganglion cells, in the lamprey retina, despite the existence of only 1 melanopsin gene in the lamprey. In contrast, melanopsin was almost specifically distributed to retinal ganglion cells in the hagfish retina. Furthermore, we found that the melanopsin-expressing horizontal cells connected to the rhodopsin-containing short photoreceptor cells in the lamprey. Taken together, our findings suggest that in cyclostomes, the global distribution of melanopsin in retinal cells might not be related to the melanopsin gene number but to the extent of retinal contribution to visual function.

## Introduction

Melanopsin (also referred to as Opn4) is an opsin-based pigment that is found in various deuterostomes, including echinoderms, cephalochordates, and vertebrates [1], [2]. Molecular phylogenetic analyses suggest that melanopsin is an ortholog of the Gq-coupled invertebrate visual opsin [3], which drives the Gq-mediated phototransduction cascade in protostome rhabdomeric photoreceptors [1], [4]–[6]. The finding that amphioxus melanopsin colocalizes with the Gq-type G protein in rhabdomeric photoreceptor cells [7] and activates Gq *in vitro* [8] also suggests an evolutionary linkage between melanopsin and the invertebrate visual opsins, both of which underlie the depolarizing light responses of rhabdomeric photoreceptors [9]–[13].

Several lines of evidence have revealed the biological functions of melanopsin in mammals. Melanopsin localizes to a small number of intrinsically photosensitive retinal ganglion cells (ipRGCs) and underlies their light-dependent depolarization [11]–[13]. The light responses of ipRGCs are involved in the regulation of non-image-forming tasks, including photoentrainment of circadian clocks and pupillary light reflexes [14]–[18]. Conversely, previous studies revealed that most nonmammalian vertebrates possess multiple melanopsin genes in various types of retinal cells [19]–[23]; however, the function of melanopsins in nonmammalian vertebrates remains uncertain.

**Figure 1:**
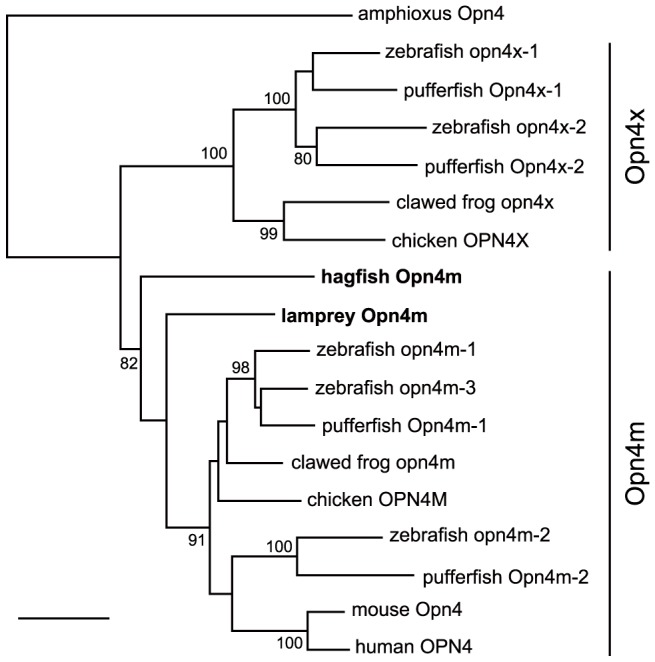
Phylogenetic position of the lamprey and hagfish melanopsins. Both the lamprey and hagfish melanopsins belong to the Opn4m group. The bootstrap probabilities >80% were indicated. Scale bar, 0.1 substitutions per site. The accession numbers of the sequences are as follows: amphioxus Opn4, AB205400; chicken OPN4M, AY882944; chicken OPN4X, AY036061; clawed frog opn4m, XP002937616; clawed frog opn4x, AF014797; hagfish Opn4m, AB932627; human OPN4, AF147788; lamprey Opn4m, AB932626; mouse Opn4, AF147789; pufferfish Opn4m-1, XP_003963814; pufferfish Opn4m-2, XP_003976773; pufferfish Opn4x-1, XP_003965597; pufferfish Opn4x-2, XP_003974868; zebrafish opn4m-1, GQ925715; zebrafish opn4m-2, AY078161; zebrafish opn4m-3, GQ925717; zebrafish opn4x-1, GQ925718; zebrafish opn4x-2, GQ925719.

Vertebrate melanopsins can be phylogenetically divided into 2 types, the Opn4m and the Opn4× [22]. Mammals possess only the *Opn4m* gene, indicating that the *Opn4×* gene was secondarily lost during the evolutionary process that led to the mammalian lineage [22], [24]. In contrast, most nonmammalian vertebrates possess both types of melanopsin genes (see Fig. 1). In zebrafish, 5 melanopsin genes, 2 *Opn4×* and 3 *Opn4m* genes, are expressed in photoreceptor, horizontal, bipolar, amacrine and ganglion cells [19]. In the chicken retina, 2 melanopsin genes are widely expressed in various types of cells, with the exception of the retinal pigment epithelium and Müller cells [21], [23], [25]. The distribution of multiple melanopsins in various types of retinal cells suggests a more complicated biological function of melanopsin in nonmammalian vertebrates compared with those observed in mammals.

We reported previously that only 1 *Opn4m* gene is present in the genome database of the cyclostome sea lamprey (*Petromyzon marinus*) [1], which is among the most primitive nonmammalian vertebrates. The number of melanopsin genes in the lamprey is quite different from that detected in other nonmammalian vertebrates, and is similar to that found in mammals [22]. Therefore, because only 1 *Opn4m* gene was identified in its genome database and given the phylogenetic position of cyclostomes at critical stages in vertebrate evolution, lamprey is a suitable animal for investigating melanopsin functions in nonmammalian vertebrates.

In this study, we investigated the molecular properties and distribution of melanopsin in 2 cyclostomes, the lamprey (*Lethenteron camtschaticum*) and hagfish (*Eptatretus burgeri*). The lamprey possesses developed eyes that underlie visual functions, including image-forming vision, whereas the hagfish eyes have no lens, are buried beneath the skin, and are primarily involved in non-image-forming vision, rather than image-forming vision [26]–[28]. We identified a single melanopsin in each of these animals, and demonstrated that they have similar light-activated functionalities. In addition, we observed that melanopsin was localized to several types of retinal cells in the lamprey and primarily to ganglion cells in the hagfish. We discuss the relationship between melanopsin distribution in the retinas and the extent of the visual functions of the retina.

## Materials and Methods

### Ethics statement

This experiment was approved by the Osaka City University animal experiment committee (#S0032) and complied with the Regulations on Animal Experiments from Osaka City University.

### Animals

River lampreys (*L. camtschaticum*) were kindly provided by Prof. Satoshi Tamotsu (Nara Women's University). Hagfishes (*E. burgeri*) were commercially obtained.

### Isolation of melanopsin cDNAs

Total RNA was isolated from the eyes of the lamprey and hagfish. Total RNA was reverse transcribed to cDNA using oligo (dT) primers. These cDNAs were subsequently used as templates for PCR amplification. A partial cDNA of the lamprey melanopsin was obtained using gene-specific primers that were designed according to the genome sequence of the sea lamprey (*P. marinus*) melanopsin. A partial cDNA of the hagfish melanopsin was obtained using degenerate primers that were designed based on the conserved region of the seven transmembrane domains of vertebrate melanopsins, including the lamprey melanopsin. The sense and antisense degenerate primers used to obtain the hagfish melanopsin cDNA fragments were as follows: sense, 5′–TGGTCTGCITAYGTNCCNGARGG–3′, corresponding to the amino acid sequence WSAYVPEG; antisense, 5′–TAYTTIGGRTGIGTDATNGC–3′, corresponding to the amino acid sequence AITHPKY; and 5′–GCRTAIACDATIGGRTTRTGDAT–3′, corresponding to the amino acid sequence IHNPIVYA. The full-length cDNAs of the lamprey and hagfish melanopsins were obtained using the 5′RACE and 3′RACE systems (Invitrogen).

### Phylogenetic tree inference

Multiple alignment of the amino acid sequences of melanopsins, including the lamprey and hagfish melanopsins, was performed using the XCED software [29]. The phylogenetic tree was inferred as described previously [30]. In brief, the evolutionary distance was applied to the phylogenetic tree using the neighbor-joining method [31]. Bootstrap analysis was conducted using the method of Felsenstein [32]. The accession numbers of the DDBJ/EMBL/GenBank or Ensembl databases regarding the sequences used in the analyses are provided in the legend to Fig. 1.

### Photopigment expression and spectrophotometry

The cDNAs of C-terminal-truncated melanopsin (387 amino acids in lamprey melanopsin) were tagged with the monoclonal antibody rho 1D4 epitope sequence (ETSQVAPA). The tagged cDNAs were inserted into the pMT2 vector obtained from Addgene (Addgene plasmid 15896). Pigment expression in COS-1 cells and pigment purification were performed as described previously [33], [34], with some modifications. Briefly, to constitute the pigment, the expressed proteins were incubated with 11-*cis* retinal overnight. The pigments were then extracted with 1% (weight/vol) dodecyl β-d-maltoside in 20 mM HEPES buffer (pH 7.0) containing 140 mM NaCl, 20 mM Tris, 0.2% cholesterol hemisuccinate, and 10% glycerol. For purification, the pigments in the crude extract were bound to 1D4-agarose, washed with 0.05% (weight/vol) dodecyl β-d-maltoside in 20 mM HEPES buffer containing 140 mM NaCl, 1 mM Tris, 0.2% cholesterol hemisuccinate, and 10% glycerol (buffer A), and eluted with buffer A containing the 1D4 peptide. The absorption spectra of the pigments were recorded at 10°C using a Shimadzu UV-2450 spectrophotometer (Shimadzu, Japan).

### Calcium imaging assay

Full-length melanopsins of lamprey and hagfish were inserted into pcDNA3.1, and the C-terminal-truncated melanopsins of amphioxus [8] and mouse [35] were cloned into the pMT2 vector. All of the melanopsins were tagged with the monoclonal antibody rho 1D4 epitope sequence. The melanopsin expression constructs were transfected into COS-1 cells with the FuGENE HD Transfection Reagent (Promega). After overnight incubation at 37°C with 11-*cis* retinal, the cells were loaded with 5 µM Fura 2-AM (Dojindo, Japan) in Krebs–Ringer HEPES buffer (20 mM HEPES, 115 mM NaCl, 5.4 mM KCl, 0.8 mM MgCl_2_, 1.8 mM CaCl_2_ and 13.8 mM glucose, pH 7.4) for 1 h at 37°C in the presence of 0.04% Pluronic F-127 and 1.25 mM Probenecid; the cells were then rinsed with Krebs–Ringer HEPES buffer. The ratios of Fura-2 fluorescence at excitation wavelengths of 340 nm and 380 nm were measured in Krebs–Ringer HEPES buffer with 1.25 mM Probenecid using a fluorescence microscope (Olympus) and the MetaMorph software (Molecular Devices).

### Preparation of frozen sections

Lampreys and hagfish were quickly decapitated. Their eyes were immersion -fixed overnight in 4% paraformaldehyde in 100 mM sodium phosphate buffer (PB, pH 7.4) at 4°C, and cryoprotected by immersion in 100 mM PB containing 15% sucrose, which was later replaced with 30% sucrose. Finally, the eyes were embedded in OCT compound (Sakura, Japan) and 8–12- µm frozen sections were prepared at −20°C using a cryostat (HM 520; Microm International GmbH).

### *In situ* hybridization

*In situ* hybridization analyses were performed as reported previously [30], with slight modifications. In brief, digoxigenin (DIG)-labeled antisense and sense RNA probes for the lamprey and hagfish melanopsins were synthesized using a DIG RNA labeling kit (Roche Applied Science). The sections were treated with proteinase K (1 µg/mL) for 10 min, followed by hybridization with DIG-labeled RNA probes diluted in Ultrahyb-Ultrasensitive Hybridization Buffer (Ambion) at 68°C overnight. The probe was detected on the sections by incubation with an alkaline phosphatase (AP)-conjugated anti-DIG antibody (1∶1000, Roche Applied Science), followed by a blue 5-bromo-4-chloro-3-indolyl phosphate/nitro blue tetrazolium color reaction.

For combined *in situ* hybridization/immunohistochemistry of the lamprey retinas, the sections were treated without proteinase K. After the hybridization step, the sections were incubated with a mixture of the AP-conjugated anti-DIG antibody and the anti-lamprey melanopsin antibody (1∶4500) (see below) overnight at 4°C. The sections were then incubated with Alexa Fluor 488 anti-rabbit IgG (1∶500, Molecular Probes) for immunohistochemical detection, followed by incubation with the fluorescent substrate 2-hydroxy-3-naphthoic acid-2′-phenylanilide phosphate/4-chloro-2-methylbenzene diazonium hemi-zinc chloride salt (HNPP/Fast Red TR, Roche Applied Science), for visualization of *in situ* hybridization.

### Preparation of an antibody specific to the lamprey melanopsin

A rabbit polyclonal antibody to the lamprey melanopsin was prepared against the N-terminal peptide sequence MEEGSMLFGVHAEPGNYSL. The specific immunoreactivity of the antibody was examined using lamprey melanopsin-expressing HEK293 cells using a method described previously [36]. Lamprey melanopsins were detected in cultured cells using the anti-melanopsin antibody (1∶4500) and the rho 1D4 antibody (hybridoma culture fluid) (Fig. S1).

### Immunohistochemical analysis of lamprey melanopsin

The sections were incubated overnight at 4°C with the anti-lamprey melanopsin antibody (1∶4500) and the anti-transducin antibody (1∶500; TF15; CytoSignal). The sections were subsequently incubated with Alexa Fluor 488 anti-rabbit IgG and Alexa Fluor 594 anti-mouse IgG (1∶500; Molecular Probes) for 5 h at room temperature.

### Retrograde labeling

Retrograde labeling was performed as described previously [37], with slight modifications. The neuronal tracer neurobiotin (Vector Laboratories) was applied to the optic nerves of the lamprey and hagfish eyes. These eyes were incubated in oxygenated Ringer solutions for lamprey [37] and for hagfish [38] overnight at 4°C, and fixed in 4% paraformaldehyde in 100 mM PB. To visualize neurobiotin, frozen sections of the lamprey and hagfish eyes were incubated with Alexa Fluor 594-conjugated and Alexa Fluor 488-conjugated streptavidin, respectively, for 5 h at room temperature. Additional immunohistochemistry and *in situ* hybridization were performed as described above for the visualization of lamprey and hagfish melanopsin localization, respectively. Nuclei were stained with Hoechst 33258 (1∶3000; Dojindo, Japan) for 5 h.

For the quantification of melanopsin distribution in ganglion cells in the hagfish retina, the ratio of melanopsin-containing ganglion cells to total melanopsin-containing cells was investigated on 8 different slices of the hagfish retina. The Wilcoxon signed-rank test was used for the analysis of the ratio.

### Microscopy

Fluorescence microscopic and Nomarski (differential interference contrast) microscopic images were obtained using a Leica DM6000 B instrument (Leica Microsystems).

## Results

### Isolation of lamprey and hagfish melanopsin cDNAs

We isolated cDNAs encoding melanopsins from the eyes of the lamprey and the hagfish via PCR amplification. The molecular phylogenetic tree of melanopsins classified the hagfish melanopsin as well as the lamprey melanopsin into the Opn4m group (Fig. 1). PCR-based screening against the hagfish eye cDNAs failed to identify the *Opn4×* gene. These results suggest that the hagfish contains only 1 *Opn4m* gene, similar to the lamprey [1].

### Spectroscopic characterization and photosensitivity of the melanopsins

**Figure 2:**
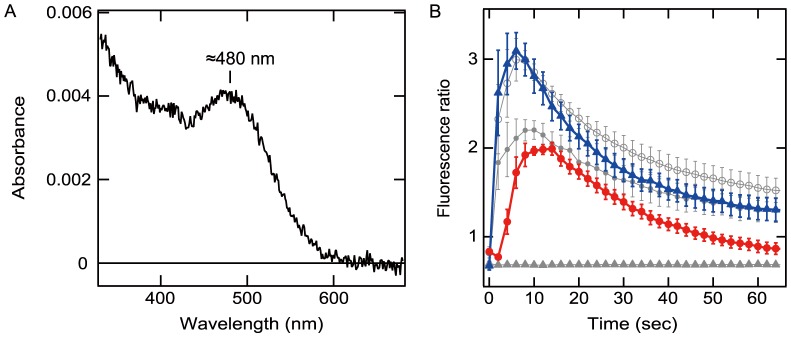
Molecular properties of the lamprey and hagfish melanopsins. A, An absorption spectrum of the dark-state lamprey melanopsin. The absorption maximum is at approximately 480 nm. B, Light-induced transient Ca^2+^ increases in lamprey (blue solid triangles), hagfish (red solid circles), amphioxus (gray solid circles) and mouse (gray hollow circles) melanopsin-expressing cells were determined by using the Ca^2+^ indicator dye Fura-2. The cells were irradiated with blue light for 500 ms immediately after the first measurement point. The mock-transfected cells exhibited no responses (gray solid triangles). Full-length melanopsins of lamprey and hagfish and C-terminal-truncated melanopsins of amphioxus [8] and mouse [35] were used (see materials and methods section for details). Each error bar represents the average value of 12 cells and indicates the standard deviation.

We first investigated the spectroscopic properties of the cyclostome melanopsins. We successfully purified lamprey melanopsin from melanopsin-expressing COS-1 cells following reconstitution with 11-*cis* retinal chromophore, although we were unable to obtain a purified hagfish melanopsin. The absorption maximum of the lamprey melanopsin was approximately 480 nm (Fig. 2A), indicating that it is a blue light-sensitive pigment and has spectroscopic properties that are similar to the mammalian ones [35], [39]. We subsequently investigated the light-dependent functionalities of the lamprey and hagfish melanopsins by Ca^2+^ imaging assay using Fura-2. In cultured cells that expressed lamprey or hagfish melanopsin, blue light stimulation induced a remarkable increase in intracellular Ca^2+^, which subsequently decreased to nearly baseline levels in a manner that was similar to that observed in the mouse and amphioxus melanopsin-expressing cells, in which light irradiation elevates Ca^2+^ levels via the activation of the Gq-type G protein [39], [40] (Fig. 2B). These results indicate that, in the cultured cells, the lamprey and hagfish melanopsins formed functional pigments with the retinal chromophore.

### Distribution of melanopsin in the lamprey retina

**Figure 3:**
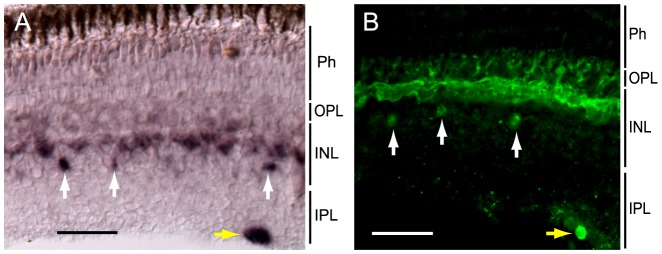
Localization of melanopsin in the lamprey retina. Localization of melanopsin visualized by *in situ* hybridization with an antisense probe for melanopsin (A) and immunohistochemistry with an anti-melanopsin antibody (B) in the lamprey retina. Melanopsin expression is observed in the inner horizontal cells of the INL and in other cells in the proximal region of the INL (white arrows) and IPL (yellow arrows). Ph, photoreceptor cell; INL, inner nuclear layer; IPL, inner plexiform layer; OPL, outer plexiform layer. Scale bar, 50 µm.

Next, we investigated the localization of melanopsin in the lamprey retina. Although only 1 *Opn4m* gene was found in the lamprey, *in situ* hybridization revealed that melanopsin was expressed in horizontal cells and in other types of cells in the inner nuclear layer (INL), as well as in the inner plexiform layer (IPL), of the lamprey retina (Fig. 3A).

We also conducted immunohistochemical analyses to investigate melanopsin distribution in the retina using an antibody against the N-terminal region of the lamprey melanopsin, which reacted with the lamprey melanopsin (Fig. S1). Immunostaining showed that melanopsin was widely distributed in the inner horizontal cells and other types of cells in the proximal region of the INL and IPL (Fig. 3B), which was consistent with the localization profile revealed by *in situ* hybridization (Fig. 3A and Fig. S2).

**Figure 4:**
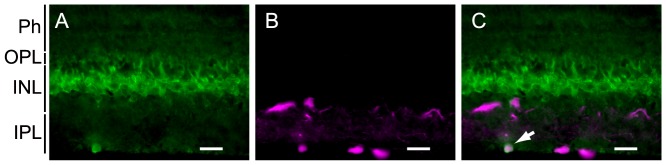
Immunohistochemical localization of melanopsin to ganglion cells identified by retrograde tracing in the lamprey retina. Melanopsin-expressing cells were immunohistochemically stained with the anti-melanopsin antibody (A, green) and ganglion cells were labeled by retrograde tracing with neurobiotin (B, magenta). A merged image reveals that melanopsin is expressed in ganglion cells (C, white arrow). Ph, photoreceptor cell; INL, inner nuclear layer; IPL, inner plexiform layer; OPL, outer plexiform layer. Scale bar, 25 µm.

In mammals, a melanopsin gene is expressed in a subset of ganglion cells [12], and therefore we then investigated whether melanopsin was expressed in the ganglion cells of the lamprey retina. Double staining using the anti-melanopsin antibody and a retrograde tracer applied to the optic nerve revealed that melanopsin was detected in the retrograde tracer-stained ganglion cells located at the IPL boundary (Fig. 4). The melanopsin-expressing cells in the INL did not overlap with the retrograde tracer-stained ganglion cells (Fig. 4), suggesting that the melanopsin-expressing cells located in the proximal region of the INL are not ganglion cells; rather, they might be amacrine or bipolar cells, according to previous morphological reports [41].

### Distribution of melanopsin in the hagfish retina

**Figure 5:**
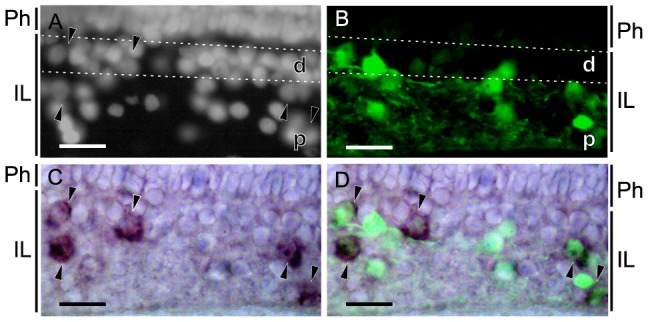
Melanopsin-expressing ganglion cells in the hagfish retina. (A) The central region of the hagfish retina stained with Hoechst consists of 2 layers, the photoreceptor (Ph) and inner layers (IL), which are further divided into two regions, the proximal region (p) and the distal region of the inner layer (d). The ganglion cells were stained by retrograde tracing (B, green), and melanopsin-expressing cells were stained by *in situ* hybridization (C, black arrowheads). Approximately 80% of melanopsin-expressing cells (75%, *p*<0.05, Wilcoxon *t*-test) were stained by retrograde tracing in the merged image (D, black arrowheads). Scale bar, 25 µm.

We then investigated the distribution of melanopsin in the retina of another cyclostome, the hagfish, which is suggested to be primarily involved in non-image-forming vision, rather than in image-forming vision [26]–[28]. Retrograde tracing clearly labeled cells in the proximal and distal regions of the inner layer, respectively (Fig. 5A and B). *In situ* hybridization revealed that melanopsin was localized to cells in both the proximal and distal regions of the inner layer (Fig. 5C), and most of the melanopsin-expressing cells were also stained by the retrograde tracing with neurobiotin (Fig. 5D). These findings suggest that the Opn4m-type melanopsin is primarily expressed in the ganglion cells of the hagfish retina, which mainly underlies non-image-forming vision, rather than image-forming vision.

### Histological connection between melanopsin-expressing horizontal cells and photoreceptor cells in the lamprey retina

**Figure 6:**
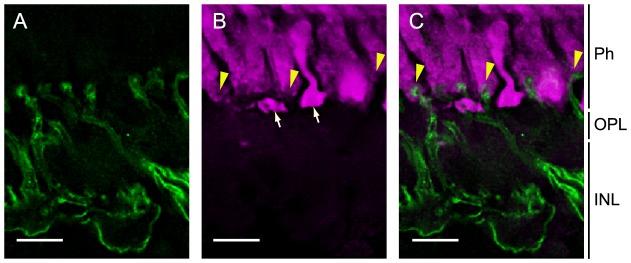
Immunohistochemical characterization of the connection between melanopsin-expressing horizontal cells and photoreceptor cells in the lamprey retina. Melanopsin-expressing horizontal cells (A, green) and two types of photoreceptor cells (B, magenta) are stained with the anti-melanopsin antibody and anti-transducin antibody (TF15), respectively. The terminals of short photoreceptor cells (B, yellow arrowheads) and long photoreceptor cells (B, white arrows) are shown. The connections between melanopsin-expressing horizontal cell dendrites and photoreceptor cell terminals are at the end of the short photoreceptor cell (C, yellow arrowheads). Ph, photoreceptor cell; INL, inner nuclear layer; OPL, outer plexiform layer. Scale bar, 10 µm.

It is well known that horizontal cells of teleost, chicken, and mammalian retinas regulate the membrane potentials of photoreceptor cells via negative feedback; therefore, we analyzed immunohistochemically the connection between melanopsin-expressing horizontal cells and photoreceptor cells, to obtain insights into the biological meaning of the global distribution of melanopsin in the lamprey retina. It has been reported that the lamprey has short and long photoreceptor cells, which are distinguishable by the terminals that appear in distinct layers [42]. Therefore, we analyzed to which type of photoreceptor cells the melanopsin-expressing horizontal cells were connected. We performed double immunostaining using the anti-melanopsin antibody and the anti-transducin antibody (TF15), which stained both types of photoreceptor cells [43] (Fig. 6 and Fig. S3). The dendrites of the melanopsin-expressing horizontal cells that invaginated into the photoreceptor cell terminals were stained with the anti-transducin antibody (Fig. 6C and Fig. S3A), and these connections were observed close to the scleral region, where terminals of the short photoreceptor cells were located (Fig. S3B). These results suggest that the melanopsin-expressing horizontal cells were connected to the short photoreceptor cells.

## Discussion

In this study, we isolated mammalian-like melanopsins (Opn4m) from the lamprey and hagfish. The fact that the lamprey has only 1 *Opn4* gene in its genome has raised 2 possibilities. First, the common ancestor of cyclostomes had only 1 *Opn4* gene and *Opn4m* and *Opn4×* split after the cyclostome–gnathostome split in the gnathostome lineage. Second, it originally had *Opn4m* and *Opn4×* and lost *Opn4x*. However, our phylogenetic analysis revealed that the split of the Opn4m and Opn4× types occurred before the cyclostome–gnathostome split [1], which supports the latter scenario. In addition, PCR-based screening against the hagfish eye cDNAs failed to identify the *Opn4×* gene, suggesting that the hagfish also contains only the *Opn4m* gene, similar to the lamprey. The result also supports the idea that the ancestral cyclostome had *Opn4m* and *Opn4×* genes and secondarily lost the *Opn4×* gene.

We report the absorption spectrum of lamprey melanopsin, with its absorption maximum at approximately 480 nm, which was similar to those of other melanopsins. We also showed light-dependent Ca^2+^ increases in the lamprey and hagfish melanopsin-expressing cultured cells, similar to those observed in mouse melanopsin-expressing cells [39], [40] (Fig. 2). Taken together with previous observations [19], [35], [39], [40], these findings suggest that cyclostome melanopsins have similar characteristics to mammalian ones.

Our *in situ* hybridization and immunohistochemical analyses revealed that strong melanopsin expression was observed in a large number of inner horizontal cells of the lamprey retina. The dendrites of the melanopsin-expressing horizontal cells formed contacts with the rhodopsin-containing short photoreceptor cells (Fig. 6). It is widely accepted that a principal function of retinal horizontal cells in various vertebrates is the provision of negative feedback to photoreceptor cells [44]–[47]. Therefore, lamprey melanopsin in horizontal cells may be involved in the regulation of negative feedback from the horizontal cells to the rhodopsin-containing short photoreceptor cells in the lamprey retina. In catfish and goldfish, it was reported that some cone horizontal cells, which might contain melanopsin, exhibited inward calcium ion currents as a response to light [48]. In addition, in a cyprinid teleost, the roach (*Rutilus rutilus*), an electrophysiological study suggested that a subset of horizontal cells are intrinsically photosensitive and light-dependently depolarized [49]. These observations suggest that the lamprey horizontal cells containing melanopsin might depolarize upon light absorption. Therefore, one possibility for the function of melanopsin is the depolarization of the melanopsin-containing horizontal cells, which inhibits or cancels the hyperpolarization responses of horizontal cells that are triggered by light-dependent hyperpolarization of the photoreceptor cells, and, consequently, regulates the negative feedback to the photoreceptor cells. Accordingly, the possible depolarization of horizontal cells caused by melanopsin light absorption may contribute to membrane potential regulation in the rhodopsin-containing short photoreceptor cells, as a modulation of visual function.

Nonmammalian vertebrates, such as fish and chicken, possess multiple *Opn4* genes that are widely expressed in most retinal cell types, with the exception of retinal pigment epithelial and Müller cells [19]–[21], [23]. In contrast, mammals possess a single melanopsin gene that is limitedly expressed in ganglion cells. In this study, we found that in the lamprey, melanopsin was distributed among various types of retinal cells, including horizontal cells and ganglion cells, although the lamprey has only 1 mammalian-like melanopsin gene. Therefore, these findings indicate that there is no significant relationship between the number of melanopsin genes and the multiplicity of melanopsin-expressing cell types. Rather, our results highlighted the fact that, regardless of the number of melanopsin genes, melanopsin(s) can be distributed in various retinal cells in nonmammalian vertebrates, particularly in horizontal cells, which functionally contact with photoreceptor cells. In other words, melanopsin may contribute to the regulation of various visual functions, including both image-forming and non-image-forming visions in nonmammalian vertebrates, such as lampreys. Conversely, in the hagfish retina, which is mainly involved in non-image-forming vision, melanopsin was primarily distributed to the ganglion cells. Although we compared the distribution of melanopsin in only two cyclostome retinas, our findings allow us to speculate that the global distribution of melanopsin to various kinds of retinal cells is not correlated with the number of melanopsin genes, but might be related to the extent of the retinal contribution to visual function, especially the contribution to image-forming vision in cyclostomes.

## Supporting Information

Figure S1 **Immunoreactivity of the antibody against lamprey melanopsin.** HEK293 cells expressing lamprey melanopsin were immunostained with the anti-lamprey melanopsin antibody (A) and rho 1D4, a monoclonal antibody to bovine rhodopsin C-terminal sequence, which was tagged on the lamprey melanopsin C-terminus (B). (C) A merged image showing that the antibodies labeled the same cells. (D) Nomarski image of the HEK293 cells. (E) Immunoblot profiles demonstrate that the anti-melanopsin and rho 1D4 antibodies specifically recognized an ∼43 kDa peptide (lines 1 and 2). M indicates the molecular weight standard marker (lane 3; Bio-Rad Laboratories). Scale bar, 50 µm.
(EPS)

Figure S2 **Identification of melanopsin-expressing cells by combined** ***in situ*** **hybridization and immunohistochemistry.** Fluorescence *in situ* hybridization with HNPP/Fast Red staining (A) and immunohistochemistry with anti-melanopsin antibody (B) shows melanopsin expression in inner horizontal cells (arrowheads) of the INL and in the IPL (arrows) of the lamprey retina (see Materials and Methods section for details). A merged image (C) indicates that the anti-melanopsin antibody-labeled cells (green) overlap with the melanopsin probe-stained cells (magenta). INL, inner nuclear layer; IPL, inner plexiform layer. Scale bar, 25 µm.
(EPS)

Figure S3 **Immunohistochemical image and schematic model of the connection between melanopsin-expressing horizontal cells and photoreceptor cells.** The connections between melanopsin-expressing horizontal cell dendrites and the short photoreceptor cell terminals were immunohistochemically analyzed with the anti-melanopsin antibody (A, green) and anti-transducin antibody (A, magenta). The connections are indicated by arrowheads in the merged image (A). A schematic drawing (B) shows that the terminals of short photoreceptor cells are closer to the scleral region than are those of long photoreceptor cells. E, ellipsoid bodies; N, nuclei; Ph, photoreceptor cell; IHC, inner horizontal cell; INL, inner nuclear layer; LPC, long photoreceptor cell; LPC ter, long photoreceptor cell terminal; OPL, outer plexiform layer; SPC, short photoreceptor cell; SPC ter, short photoreceptor cell terminal. Scale bar, 25 µm.
(EPS)
